# Therapeutic and Preventive Use of Video Games in Child and Adolescent Psychiatry: A Systematic Review

**DOI:** 10.3389/fpsyt.2020.00036

**Published:** 2020-02-06

**Authors:** Darius Zayeni, Jean-Philippe Raynaud, Alexis Revet

**Affiliations:** ^1^ Service Universitaire de Psychiatrie de l’Enfant et de l’Adolescent, CHU de Toulouse, Toulouse, France; ^2^ UMR 1027, Inserm, Université Toulouse III, Toulouse, France

**Keywords:** child and adolescent psychiatry, video game, exergame, serious game, therapy

## Abstract

**Background:**

Over the past decade, the use of commercial video games and serious games has developed in child and adolescent psychiatry. These games may become relevant alternatives or adjuncts to traditional psychotherapy, providing that their effectiveness is properly established. The purpose of this literature review was to evaluate the effectiveness of serious games and commercial video games in the treatment or prevention of psychiatric disorders in children and adolescents.

**Methods:**

Medline’s database was used to search articles published between January 2012 and July 2019. The following keywords were used for this search: “Video games” OR “Active video game” OR “serious gaming” OR “Serious game” OR “Exergame” AND “Child mental disorder” OR “Adolescent” OR “Child” AND “Therapy” OR “Prevention”. Only comparative studies which targeted interventions on children and adolescents suffering from psychiatric disorders were included.

**Results:**

Twenty-two studies, focusing on a wide range of psychiatric conditions, met our inclusion criteria's: 14 evaluated serious games and 8 commercial games. All studies were randomized controlled trials but only two studies compared the intervention game to psychotherapeutic gold standard; other studies used a no-intervention control group or an alternative game as the control group. Eighteen studies reported significant improvements on the symptoms and test scores targeted.

**Conclusion:**

Serious games and commercially available video games can be an effective trajectory for psychotherapy in child and adolescent psychiatry. However, there is a lack of longitudinal studies which assess the sustained effects of these games, and standards for proper evaluation of their effectiveness are missing.

## Introduction

Video games are a major form of entertainment, specifically among children and adolescents (1, 2). With the release of America's Army in 2002, the first serious game that reached important public awareness, there has been a growing body of research considering video games as a tool to amplify motivation and learning, giving them wider applications than entertainment (3–5).

Serious games are designed to teach, through a ludic medium, a wide range of concepts and skills that can be used outside the virtual environment. Michael Zyda defines them as “a mental contest, played with a computer in accordance with specific rules, that uses entertainment to further government or corporate training, education, health, public policy, and strategic communication objectives” (6). In this study we focused on serious games that use video game design as ludic medium in contrast with role playing games or board games. In order to provide a comprehensive overview of video games' use in child and adolescent psychiatry, we also focused on commercially available video games used in a therapeutic or preventive context.

Serious games are currently used, inter alia, in therapeutic education, prevention, treatment of various medical conditions, rehabilitation, or as an educational tool for healthcare professionals (7–9). To achieve these goals, serious games use traditional video game design to create immersive and entertaining games, but they also include learning theories and methodologies based on empirical research to assure ideal learning conditions and maximize learnings. For instance, the use of a meaningful context by creating a graphic environment, rules and scenario that serves learning objectives, amplifies learning and new knowledge application to real life (10). Cognitive load theory suggest that removing cognitive load by introducing gradually difficult tasks and building a simple and user friendly interface, contributes to maintain a high level of attention through the entire game (11). Finally, serious games are designed to include evidence-based therapeutic techniques and are often based on cognitive behavioral therapy (CBT), cognitive remediation or neuropsychological theories.

Another classical distinction is between sedentary and active video games, also known as exergames. These games promote physical movement and require from the player to practice strength, balance and flexibility activities (12). To translate the players' movements into a virtual movement these games require motion controllers, boards using pressure sensors such as the Wii balance board, or cameras such as the Microsoft Kinect. These video games have raised a lot of clinical interest over the past decade, both as commercially available video games (used within an evidence-based neuropsychological framework), or specifically designed serious games. Exergames have been used to train many executive functions (13, 14), and, as pointed out by a recent meta-analysis (15), they have been used to improve cognitive functioning in neurological disabilities of older adults or school-aged children affected by developmental disorders (16, 17). They also increase daily physical activity in populations such as children with autism, without whom may have difficulty accessing collective sports (18). Such games are easily accessible, affordable and widely available for the general population and medical practitioners.

The use of video games (both as serious games and commercially available video games) in child and adolescent psychiatry can be specifically suited for many reasons. Within this age range, there is a need for therapeutic alternatives since medication options are often limited (19). Children and adolescent are drawn to this medium, so adherence and engagement to treatment can be amplified. Also, learning new abilities in the context of standard therapies such CBT or cognitive remediation can be a prolonged and fastidious process in this population (20), in particular in children and adolescents with attention disorders. By combining within a video game evidence-based therapy techniques with learning theories, serious games can make this learning process less intrusive and more enjoyable for patients, through the use of a familiar medium that feels safe, gratifying and immersive, thus helping child and adolescent to maintain their attention and motivation during extensive training sessions. Like other form of play, playing video games in a therapeutic context is a proper way for children to learn how to overcome real life issues, since developmental psychology theories highlight the fact that playing is a fundamental way in which children learn, experiment with different social experiences and emotional consequences or reproduce real-life conflicts in order to work out ideal resolutions (21, 22).

Many serious games have been designed to target symptoms of a broad range of children and adolescents' disorders. Previous literature reviews and meta-analyses on serious games highlighted the lack of research focusing on the effectiveness of these games, but also the lack of comparative studies and of follow-up evaluations (evaluations after last training session) (23–25). Moreover, most of the studies lacked statistical power due to small sample size and showed no generalization regarding acquired knowledge outside of the virtual world or lack of far transfer (near transfer occurs when the training context is similar to the application context and far transfer occurs when these contexts are remote from one another). The lack of far transfer is a recurrent concern about serious games and computerized training programs (26). For instance, while many computerized neurocognitive training programs offered to train working memory in order to reduce ADHD symptoms or reading disorder symptoms, a meta-analysis from 2013 showed that these programs improved working memory skills but training had no impact on daily life functioning and symptoms (27). Authors suggested that this lack of far transfer could be linked to the predictive and repetitive tasks, lack of context, storyline, and behavioral strategies (e.g., reinforcement, immediate performance feedback from a mentor, goal setting through missions, modeling, social support, and comparison) offered by these computerized training program (28).

An additional area of research surrounding video games has been the study of the cognitive impact of playing commercially available video games. These games often require many skills and a high tolerance to frustration, since most levels need to be practiced over and over in order to get the required skills to finally succeed (29). A recent meta-analysis concluded that action video games robustly enhance the domains of top-down attention and spatial cognition (30). A systematic literature review of experimental studies found that video games improves emotion regulation (31). Starcraft and other tower defense games can be used to foster acquisition of mathematical competence such as problem solving, while Minecraft can be used to teach mathematics, and Assassin's Creed to teach history (32–34). These games have been used in psychotherapy with children and adolescent since the early 90's (35), in order to evaluate children's cognitive processing style or to build a therapeutic relationship (36). A literature review gathered studies evaluating the cognitive impact of video games by genres, in order to set indications in therapies for each type of games (37). For instance, role playing video games (games that encourage the player to have a high level of identification to their own characters or avatars in game) can be used by psychotherapist to identify or test object-relations of patients or can be used within a CBT to challenge self-schema and generate positive alternatives.

Whilst considering that this domain of research is promising, previous authors have highlighted the need for comparative evaluations of these different types of games and regular evaluations of their effects in these indications (38). Therefore, the aim of this literature review was to evaluate the effectiveness of serious games and commercially available games in the treatment and prevention of psychiatric disorders in children and adolescents.

## Methods

### Search Methodology

The methodology of this study was in accordance with the Preferred Reporting Items for Systematic Reviews and Meta-Analyses (PRISMA) (39).

Medline's database was first used to systematically search articles published between January 2012 and July 2019. The following search algorithm was used for the search: (Video games [MeSH] OR serious game OR active video game OR serious gaming OR exergame) AND (child mental disorder [MeSH] OR (adolescent [MeSH] OR child [MeSH] AND (therapy [MeSH] OR prevention))).

In addition, we handed searched references lists of identified articles and pertinent reviews for additional studies. We also did cross searches in MEDLINE and Web of Sciences by using the names of the researchers who were authors on studies found in our first selection process.

### Inclusion Criteria

We selected comparative studies reporting the use of commercial video games or serious games in the treatment or prevention of child and adolescent psychiatric disorders. Titles and abstracts were reviewed by two independent investigators (DZ and AR), and all relevant articles were then reviewed in full-length.

We only included recent studies (since 2012) in order to evaluate the current state of research on the therapeutic and preventive use of serious games and commercial video games.

### Exclusion Criteria

We excluded computerized-based therapy that does not include storytelling or serious game design elements (virtual reality and computerized cognitive training).

Virtual reality games would be a promising area to explore; however, the development of such technology is still relatively expensive and is considered to be complex, which explains why, most of the time, they do not include storytelling and other essential elements required for serious games. Whilst they cannot be considered as serious games, they can, however, be used as important training tools. Within the studies carried out so far, the evaluation of virtual reality for child and adolescent psychiatry has included a minimal number of patients and rarely had control groups. This makes the results of studies focused on virtual reality difficult to interpret, which explains why we excluded them from this literature review.

Computerized cognitive trainings were excluded because they are not considered to be games, since they do not include a scenario and are not thought to be playful, thus lacking the potential positive effect on attention and motivation.

Studies evaluating the effects of serious games for diagnosis of child and adolescent psychiatric disorders where excluded, as well as serious games used in any other context than child and adolescent psychiatry (for example serious games designed to prevent child obesity or serious games used for neurorehabilitation).

Finally, studies describing serious games' development or acceptability were excluded since they do not focus on the therapeutic or preventive use of such games.

## Results

**Figure 1:**
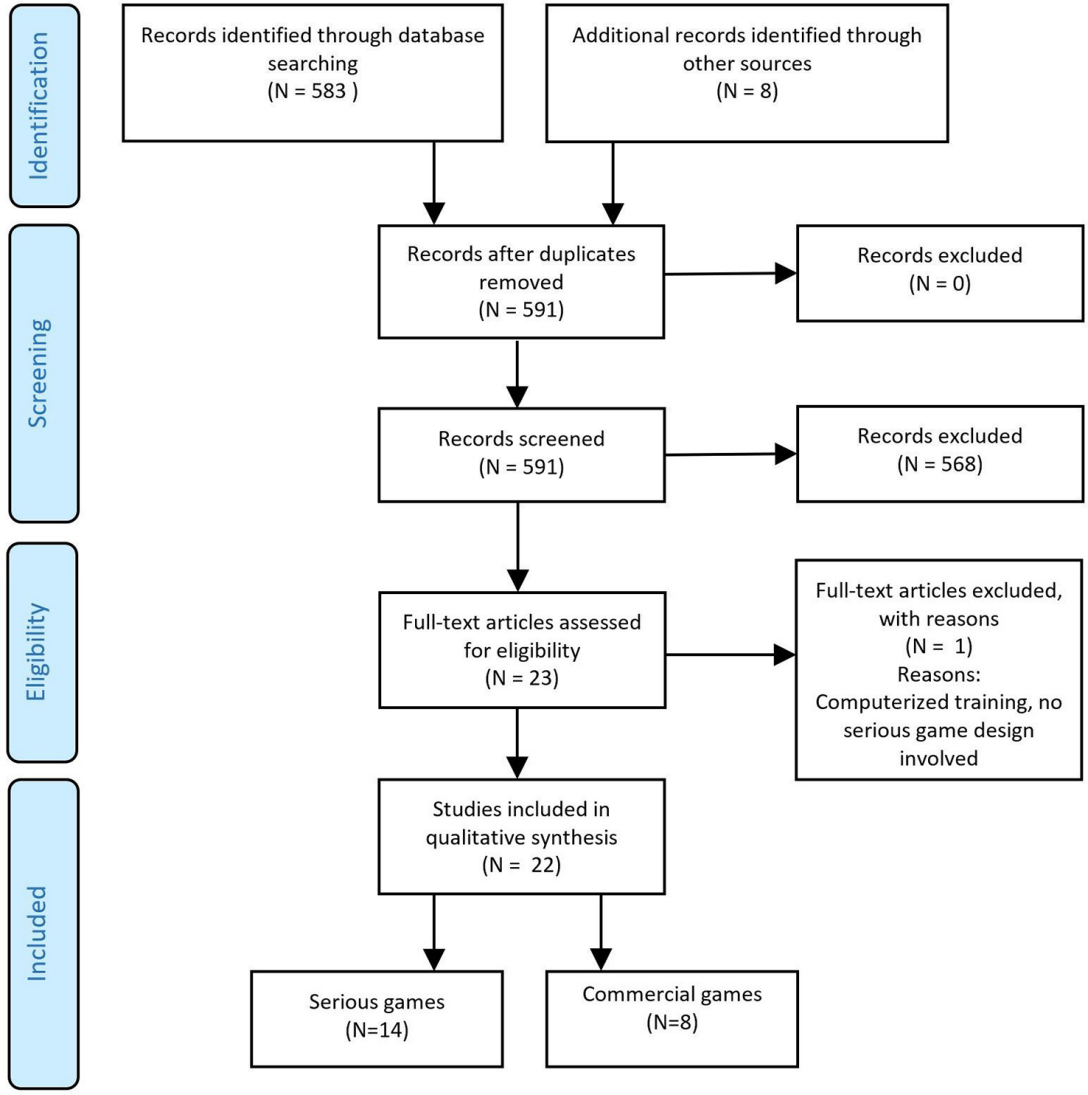
Flow diagram of the selection process.

Twenty-two studies met our inclusion criteria, 14 evaluating serious games and eight evaluating commercially available games, five of which were exergames (**Figure 1**). All studies were randomized controlled trials (RCTs), with sample sizes varying from 32 to 540 participants for studies evaluating serious games and from 18 to 100 participants for studies evaluating commercially available video games.

Studies targeted a wide range of child and adolescent psychiatric conditions including Attention Deficit Disorder with Hyperactivity, Autism Spectrum Disorder, Anxiety, Developmental Coordination Disorder and dyslexia. Eighteen studies reported significant improvement on the targeted symptoms and test scores.

Eighteen studies reported a generalization of knowledge acquired in game into daily lives. Only two studies compared the intervention game to its equivalent psychotherapeutic face-to-face therapy, (40, 41), and one study compared the game to treatment as usual (42). The other studies used a no-intervention control group and/or a different game as the control group. Last evaluation (follow-up) took place one week to 18 months after the intervention and studies evaluating commercially available video games lacked any follow-up evaluation.

Serious games used in these studies were designed to include empirical-based therapeutic framework (mostly CBT and cognitive remediation). Serious games were well-accepted by participants and succeeded in implementing core elements of serious game design in order to maintain a high level of motivation throughout the therapy process.

Detailed results are presented in **Table 1**.

**Table 1:** Characteristics of studies included in the literature review.

Author, year, reference	Game (type and platform)	Diagnosis	Target symptoms	Study design	Population (sex, age)	Estimated playing time	evaluations after last training session	Main results	Generalization of training
David et al., 2019 (40)	REThink (SG, IPAD, online)	Healthy subjects	Prevention of emotional disorders, psychological resilience.	RCT comparing three groups: one group had a face-to-face CBT intervention based on the REBE program (Rational Emotive Behavior Education); a group played the SG REThink which is based on the same program; and a waitlist group	SG: 54 REBE group: 55 waitlists: 56 10–16 y/o	7 sessions of 50 min each	No (follow-up results are presented in a different study)	Participants in the REThink group presented a significant reduction in overall emotional symptoms, with a moderate effect size, and decrease in depressive mood, with a large effect size. REThink also had a positive impact on the ability to regulate emotions with a significant effect on emotional awareness and ability for emotional control.	Far transfer
Schoneveld et al., 2018 (43)	MindLight (SG, PC, neurofeedback)	Children with elevated anxiety symptoms	Anxiety prevention	Two-armed randomized controlled non-inferiority trial comparing the SG to a CBT program aiming at preventing anxiety (coping cat)	EG: 86 CBT group: 88 7–12 y/o	1-hour session per week, during 5 weeks, school-based	Last evaluation 6 months after training	MindLight is as effective as CBT in the prevention of anxiety and showed a larger decrease in child reported personalized anxiety symptoms at post-test and 6-month follow-up. Three and 6-month follow-up assessments indicated that improvements were sustained based on both child and parent reports of anxiety measures.	Far transfer
DeSmet et al., 2018 (44)	Friendly Attac (brief SG intervention, PC, offline)	All 8th grade classes from two schools in Flanders	Increase in positive bystander behavior and reduction in negative bystander behavior, to prevent and reduce cyberbullying	Cluster RCT, the control condition received the intervention after the follow-up measurement was completed	EG: 134 CG: 115 13–14 y/o	One session of less than 30 min	Yes, 4 weeks	Significant improvements in self-efficacy, prosocial skills, and the intention to act as a positive bystander (increased witnessing of cyberbullying incidents); an increase in quality of life was also noticed; no significant effects were found for predictors of negative bystander behavior; no effects were found on behavior itself, bullying or cyberbullying prevalence.	Far transfer
Perry et al., 2017 (45)	SPARX-R (SG, PC, online)	Adolescents enrolled in their final year of secondary school, no exclusion criteria	Preventing the development or exacerbation of depressive symptoms, prior to a stressful event (final secondary school exams)	Cluster RCT: 2 parallel arms consisting of an experimental condition (SPARX-R) and an attention-matched control condition providing no direct mental health content (lifeSTYLE online intervention)	EG: 242 CG: 298 Mean age of 16.7 (SD 0.51) years	7 sessions of 20 to 30 min. 1 or 2 sessions per week, School-based	Yes, at 18 months	Less depressive symptoms post intervention and at 6-month follow-up but not at 18 months post-baseline.	Far transfer
Boendermaker et al., 2017 (46)	The Fling (SG, PC, offline)	Typically developing adolescents	Behavioral control, control over alcohol use	RCT comparing three experimental groups: game training, game placebo training and nongame training	game-training: 70 (31 M; 39 F); game placebo training: 60 (37 M; 23 F); and nongame training: 55 (10 M; 45 F) 13–17 y/o	4 sessions of 10 to 15 min, spread over 4 weeks, school based	Yes, at 4 to 6 weeks	Behavioral control increased significantly between pre- and posttest, in all three conditions, including the placebo training; no significant effects of the training were found on the adolescents' drinking frequency.	Near transfer
Sanchez et al., 2017 (47)	Adventures aboard the S.S. GRIN (SG, PC, online)	Children struggling with social-emotional skills (scoring at or above the clinical cutoff on any subscale of the BASC-2)	Social literacy, social self-efficacy, social anxiety, social satisfaction, bullying victimization, bullying perpetration	RCT comparing immediate treatment condition or a wait-list control condition	IG: 33 (19 M; 14 F) CG: 36 (22; M 14 F) 7–11 y/o	9 episodes of 25 min each over 9 weeks, home based	Last evaluation 1 week after training	Increase social-emotional skills knowledge and understanding (measured by the Achieved Learning Questionnaire); measures of social anxiety, social satisfaction, and bullying victimization were also significantly improved after playing the SG; absence of significant increase in children's belief in their ability to succeed in social situations.	Far transfer
Bul et al., 2016 (48)	Plan-It Commander (SG, PC, online)	ADHD	Daily life functioning: time management, planning/organizing, and cooperation skills	20-week multisite randomized controlled crossover open-label trial design: Participants randomized to group 1 received a serious game intervention in addition to treatment as usual for the first 10 weeks and then received treatment as usual only for the next 10 weeks. Participants randomized to group 2 received treatment as usual for the first 10 weeks and crossed over to the serious game intervention in addition to treatment as usual for the subsequent 10 weeks.	N = 170 (137 M; 33 F) Group 1: 88 Group 2: 82 8–12 y/o	65 min sessions approximately 3 times per week for 10 weeks, Home-based	Yes, at 10 weeks	Significant improvement in daily life functioning across domains of time management, social skills and working memory.	Far transfer
Fridenson− Hayo et al., 2016 (49)	Emotiplay (SG, PC, online)	ASD	ER	RCT conducted simultaneously in Israel and in Sweden, in which children with ASD using the SG were compared to a waiting-list control (treatment as usual)	Israel: EG: 23; CG: 20 Sweden: EG:20; CG: 20 6–9 y/o	At least 2 h a week for 8 weeks, Home-based	Yes, at 8 weeks	Significant improvement on participant's performance on ER, body language and integrative tasks; parents also reported that their children improved their adaptive socialization.	Far transfer
Weerdmeester et al., 2016 (50)	Adventurous Dreaming Highflying Dragon (Serious Exergame, PC using Kinect sensor, offline)	ADHD	Impulsivity, inattention, hyperactivity, and motor deficiency	RCT comparing two groups: intervention group played the serious exergame, while control group played a Kinect-based commercial game (Angry Birds)	EG: 37 CG: 36 6–13 y/o	6 sessions of 15 min over 3 weeks, school-based	No	Equal improvement was found in both groups in terms of fine motor skills, no significant change was found in gross motor skills. Children who played Dragon exhibited a marginally greater improvement than control group in terms of teacher-reported symptoms	Far transfer
Scholten et al., 2016 (51)	Dojo (SG, PC, heart rate variability biofeedback)	Adolescents with subclinical levels of anxiety	Anxiety prevention	RCT comparing two groups: EG played DOJO and the CG played a commercial game (Rayman)	EG: 70 CG: 68 11–15 y/o	6 sessions over 3 weeks (2 h per week), School-based	Yes, at 3 months	Anxiety symptoms significantly decreased at follow-up in both conditions.	Far transfer
Schoneveld et al, 2016 (52)	MindLight (SG, PC, neurofeedback)	Children with elevated levels of anxiety	Anxiety prevention	RCT: comparing two groups: EG played Mindlight and the CG group played Max and the Magic Marker, a commercially available video game.	EG: 69 (30 M; 38F) CG: 66 (31 M;35F)	5 training session of 1 h, twice a week, School based	Yes, at 3 months	Children in both conditions showed significant improvements on anxiety symptoms by the 3-month follow-up, based both on child and parent reports.	Far transfer
Dovis et al., 2015 (53)	Brain game Brian (SG, PC)	ADHD combined type associated or not with ODD (oppositional defiant disorder)	WM, response inhibition and cognitive flexibility	RCT: full-active condition. In this condition WM, inhibition and cognitive-flexibility were all in training-mode Partially active condition: played the same game but the WM task was in placebo-mode Placebo group: in this condition WM, inhibition and cognitive-flexibility were all in placebo-mode	EG: 31 (25 M; 6 F) Partially active condition: 28 (22 M; 6 F) Placebo Group: 30 (M 24; 6F) Age: 8–12 y/o	25 training sessions of 35–50 min, over 5-week, Home-based	Yes, at 3 months	Improvement on measures of visuospatial STM (short term memory) and WM, inhibitory performance and interference control; significant improvement on teacher-rated ADHD behavior.	Far transfer
Murphy et al., 2014 (54)	Maze Task (SG cooperative game, PC, 2 player)	Children with impairments in social communication	Pragmatic language skills, peer cooperation	RCT comparing two group: EG and DIG: each group is composed by dyads including one child with HP and one with LP, both groups undergoes the same intervention with a time delay. The intervention consists of two procedures: one during which LP children play the game with an HP child and another during which LP children plays the game with the third author using communication training methods based on the modelling approach as developed by Palincsar and Brown (1984).(55)	EG: 16 (5 M 11 F) DIG: 16 (8 M 8F) 5–6 y/o	3 times 30 min communication intervention training sessions with the third authors and 2 sessions playing with HP children	Yes, not known	Increase on scores on the Test of Pragmatic Skills and in the use of information-seeking questions	Far transfer
Merry et al., 2012 (42)	SPARX (SG, PC, online)	Adolescents with mild-to-moderate depressive disorder	Depression symptoms, anxiety, quality of life	Randomized controlled non-inferiority trial comparing the SG (based on a CBT program) with treatment as usual.	IG: 94 (35 M; 59 F) TAU: 93 (29 M; 64 F) 12–19 y/o	7 modules of 20 to 40 min over 4 to 7 weeks, home based or in the facility where the adolescent sought help	Yes, 3 months	Clinically significant reduction in depression, anxiety, hopelessness and an improvement in quality of life with a persistence of the effects at 3 months follow up. The response and remission rates for participants in the SPARX group (66% and 44% respectively) compare favorably with other effective monotherapies, including antidepressants and cognitive behavioral therapy.	Far transfer
Łuniewska et al., 2018 (56)	Rayman Raving Rabbids (Action video game, Wii)	Polish-speaking children with dyslexia	Reading, phonological, and attentional skills	RCT comparing 3 groups: 2 training groups: AVG group playing Rayman and NAVG group playing a specifically designed game based on phonological awareness tasks; and a control group which did not participate in any video-game-based training	AVG: 27 (18 M; 9 F) NAVG: 27 (18 M; 9 F) CG: 16 (15 M 1 F) 9–13 y/o	16 training sessions of 50 min, laboratory based	Last test 1 to 18 days after training	AVG and NAVG trainings resulted in the increase of both reading accuracy and speed. However, a dyslexic control group, which did not participate in any special training, presented the same enhancement or stability of reading-related skills than the two training groups.	No generalization
Benzing et al., 2018 (57)	Shape Up (exergame, Xbox + Kinect sensor)	ADHD	Inhibition, switching and visual WM	RCT comparing two group: an acute session of exergaming condition and a control group watching a documentary about mountain running	EC: 24 (20 M; 4 F) CG: 22 (18 M; 4 F) 8–12 y/o	Single session of 15 min Home-based	No	Participants in the exergaming group performed significantly faster than those in the control group in terms of both inhibition and switching, but there was no significant difference in the accuracy of the two tasks nor in visual working memory performance.	Near transfer
Franceschini et al., 2017 (58)	Rayman Raving Rabbids (Action video game, Wii)	English-speaking children with dyslexia	Reading, phonological, and attentional skills	Comparative study comparing two matched groups of English-speaking children with dyslexia before and after they played AVG (selected action mini games from Rayman) or NAVG (selected non action mini games from Rayman).	N = 28 (20 M; 8 F) AVG: 16 NAVG: 12 7–14 y/o	9 sessions of 80 min during two weeks, Laboratory-based	Last test 1 to 3 days after training	Improvement in attention, word reading and phonological decoding efficiency after AVG training	Far transfer
Bonney et al., 2017 (59)	Wii fit (exergame, Wii + balance board)	DCD	Body function and structure (impairment), activity (activity limitations), and participation restrictions	Assessor blinded, stratified, randomized trial: participants (female adolescent) were assigned to receive either TFT or Wii training.	TFT group: 22 (22 F) Wii training group: 21 (21 F) 13–16 y/o	45 min training per week for 14 weeks, School-based	No	The two groups had significant improvement in muscular strength, motor proficiency, running and agility, predilection for physical activity and generalized self-efficacy. However, there was no difference in outcomes for the two interventions.	Far transfer
Dickinson et Place, 2016 (60)	Mario and Sonics at the Olympics (exergame, Wii, 1 to 4 players)	ASD with moderate or severe learning disability	Social functioning	Pooled subject design, with the children randomly allocated to either intervention or control group. Children allocated to the control group had the standard school physical education program. Children in the intervention group, in addition to these standard lessons, had sessions in which they used the activity game in groups of 2 to 4, under supervision.	IG: 50 (39 M; 11 F) CG: 50 (40 M; 10 F) 5–15 y/o	15 min per day, 3 times a week, 9 months period, School-based	No	Teacher-completed measures of social functioning showed that boys in the intervention group had made statistically significant improvement in their functioning when compared to controls. The number of girls in the study was too small for any change to reach statistical significance.	Far transfer
Straker et al., 2015 (61)	Range of non-violent exergames (11) provided to children (exergames, PlayStation 3 + PlayStation Eye and move and Xbox 360 + Kinect)	DCD	Physical activity, sedentary time	Crossover RCT: both groups played had an exergame intervention for 16 weeks and no exergame for 16 weeks.	N = 21 (10 M; 11 F) Group 1: 11 Group 2: 10 10–12 y/o	20 min a day, 4 to 5 days a week over a period of 16 weeks, Home-based	No	AVG intervention did not improve the physical activity or sedentary time of the participants. Participants, but not the parents, did report significantly enhanced perception of physical skills following the exergame in comparison to no exergame.	Near transfer
Hammond et al., 2014 (62)	Wii fit (exergame, Wii + balance board)	DCD	Motor proficiency, self-perceived ability and satisfaction, and parental assessment of emotional and behavioral problems	Randomized crossover controlled trial, both groups had the exergame intervention for 4 weeks and a local school-run motor intervention program (“jump ahead”) for another 4 weeks	Group A: 10 (8 M; 2 F) Group B: 8 (6 M; 2 F) 7–11 y/o	3 sessions of 10 min per week for 1 month, School-based	No	Both groups showed significant gains in motor proficiency, and in child's perception of his/her motor ability, and reported emotional well-being for many, but not all, children. Parent's ratings improved in Group A following the Wii Fit intervention but not after the “jump ahead” intervention.	Far transfer
Franceschini et al 2013 (63)	Rayman Raving Rabbids (Action video game, Wii)	Italian-speaking children with dyslexia	Reading, phonological, and attentional skills	Comparative study comparing two matched groups of Italian-speaking children with dyslexia before and after they played AVG (selected action mini games from Rayman) or NAVG (selected non action mini games from Rayman)	AVG: 10 NAVG: 10 7–13 y/o	9 sessions of 80 min during two weeks, Laboratory-based	Last test 1 to 3 days after training	Improvement in word reading and phonological decoding efficiency after AVG training	Far transfer

ADHD, Attention deficit hyperactivity disorder; ASD, Autism spectrum disorder; AVG, action video games; CBT, cognitive behavioral therapy; CG, control group; DCD, Developmental coordination disorder; DIG, delayed intervention group; EG, experimental group; ER, Emotion recognition; HP, with high pragmatic language skills; LP, low pragmatic language skills; NAVG, non-action video games; RCT, randomized controlled trial; SG, serious game; TFT, Task oriented Functional Training; WM, working memory.

## Discussion

These results highlight an overall increase in efforts to evaluate the effectiveness of serious games, as part of the treatment of psychiatric conditions in children and adolescents. Since 2015, we observed a multiplication of RCTs, with large samples variating from 32 to 540 participants, evaluating the effectiveness of serious games. Nevertheless, these studies have varied methodologies, which remain a limitation in the process of evaluating such games in a therapeutic context.

Our results are in line with those from previous meta-analyses and literature reviews on serious games, which also found no consensus on which type of control group is the best to evaluate such games (26, 64). The same issue occurs for the evaluation of commercially available video games. Three types of control groups have been used across the studies we selected: the first is a waitlist or no intervention; the second consists in the comparison to another game; and the third consists in the comparison to a gold standard psychotherapeutic equivalent (usually, face-to-face psychotherapy).

Using another game as control can reduce the accuracy of the evaluation, even more so if the control game is a commercially available one. Considering that the control game can have similar beneficial effects to the intervention game by training the same skills, this can reduce the magnitude of potential beneficial effects observed with the intervention game. This issue was present in the first evaluation of DOJO (65), where participants in both conditions (a serious game and commercial control game) showed equal improvement on anxiety symptoms. The authors highlighted that even though the control game (Rayman) was not specifically designed to reduce teenagers' anxiety, it may incorporate some mechanisms that can benefit the player's emotional development and reduce anxiety. For instance, the game is built for trial-and-error learning, which means that the player has to lose and repeat levels multiple times to sharpen his in-game skills and to do so, the individual must overcome frustration, anger and anxiety in order to win. The concern here is that most games are built on trial-and-error learning, which can have an effect on emotional development and emotion regulation (31), thereby making it complicated to use a game as a control condition. This highlights the importance of the addition of a waitlist group, in order to ensure that any beneficial effects observed are not any greater without any intervention at all. Studies evaluating the impact of commercially available video games on dyslexia fostered the crucial importance of a waitlist group. Two studies, comparing commercial action video games to non-action video games, without a waitlist group, showed improvement in reading words and phonological decoding (58, 66). Łuniewska et al. examined studies which included more participants, a wait list control group, one intervention group using the same commercially available action video game and one control group using a non-action video game specifically developed by the research team to be based on phonological awareness tasks (56). Results revealed that the two game intervention groups had the same enhancement of reading skills and that the wait list control group, which did not participate in any training, presented the same enhancement of reading skills as the two training groups. By including a no-intervention control group, this study provided evidence to support the fact that action video games do not enhance the reading or phonological skills of dyslexic children. These results imply that only exergames hold promise of efficacy among commercially available video games included in our literature review.

It is important to compare these games intending to have therapeutic benefits with the gold standard intervention in order to assess them. A meta-analysis assessing the effectiveness of serious games concluded that the best way to evaluate their effectiveness is to compare them to a group that receives no training and to a group trained using a different type of method (applied to child and adolescent psychiatry, it would be the equivalent of face-to-face therapy or gold standard therapy) (26). This was done in only two of the studies retrieved in our literature review (40, 41).

The first study designed to assess the serious game Mindlight in 2016 used a commercial video game as control group, facing the same challenge as the study evaluating the serious game DOJO: results showed significant improvement, but equal in both control group and experimental group (52). Mindlight incorporated successfully several CBT strategies within its storyline and design: attention bias modification, exposure therapy and electroencephalogram (EEG) biofeedback training. The story centers on a child, Arty, isolated in a scary haunted dark mansion trying to save his grandmother. Arty finds a magical glowing headset that teaches him (and the player) to overcome his fears by changing his state of mind, the more relaxed the player is in real life, the more the headset shines in the game, allowing Arty to pass through the dark scary mansion, and the player to progress in the game. Changes in brainwave patterns recorded by the EEG headset (i.e., reduction in relative beta power and increases in relative alpha power) have been identified as proxies of relaxation. The game incorporates exposure therapy in its design and mechanics by encouraging the player to try a variety of relaxation techniques (e.g., deep breathing, self-talk) while approaching (rather than avoid) scary enemies. Finally, the game incorporates attention bias training by including a modified dot probe task in its design. By rewarding and encouraging the player to quickly respond with a mouse click to positive stimulus and shift attention away from negative ones, the games trains the players attentional system to attend more to positive stimuli than negative ones, thus correcting attentional bias underlying the pathogenesis of childhood anxiety (41).

The second study assessing Mindlight in 2018 compared the game to 8 sessions of face-to-face CBT therapy (coping cat) in a randomized controlled non-inferiority trial (41). Results showed equal improvement in both groups in terms of self-reports and parents–teacher ratings at post-test, three and six month follow-up, thus proving that a well-designed serious game could be used as an engaging alternative way to deliver CBT to children suffering from anxiety.

Another important factor that can interfere with the accuracy of studies is the notion of transfer of acquired knowledge and skills into everyday lives. In the context of child and adolescent psychiatry, it could be possible to know if there is indeed generalization or not, through parents and teachers' evaluations and the use of self-reports. Most studies in this literature review report far transfer or generalization of knowledge into daily lives, through improvement of daily lives and reductions of symptoms on self-reports, or improvement of parents-teacher's ratings. However, follow-up evaluations were rarely made more than a month after the final training session and were not performed in RCT evaluating the effectiveness of commercially available video games in a therapeutic context. This can make it difficult to determine if the skills acquired in game and the beneficial effects in daily life will persist over time.

Finally, the design of the game itself is another factor that may influence the evaluation of the effectiveness in these studies. Serious games included in this literature review have solid theoretical bases (often CBT), but the game acts as a vector of therapy, using a variation of mechanisms to achieve this purpose. The game's design and storyline elements should be described in studies since they can be so varied, and content may be an influencing factor that needs to be considered. Important game design elements that can influence the results are immersive storylines, long terms and meaningful goals, rewards and feedbacks, adaptative levels of difficulty matching player ability (to avoid too much boredom or frustration in order to maintain a high level of engagement), and providing choices supporting player autonomy (67, 68). Overall, most studies briefly described the incorporation of these game design elements in their games and the procedures used to generalize what is learned in the game outside of it.

Previous meta-analysis from Grynszpan et al., revealed a negative correlation between duration of training and effectiveness of the intervention (25). This observation was linked to the fact that shorter intervention was supervised by a specialist whereas longer intervention (10 weeks or more) were not (participants of these studies learned how to use these technologies by themselves or with the help of their parents).

In our literature review, we found no correlation between efficacy and duration of training. Instead, few authors pointed out a correlation between efficacy and quantity of content. To maintain the engagement and the attention of the player, the game has to provide enough content and challenges throughout the entire duration of the training, otherwise training may lose its efficacy.

This limitation appeared in the evaluation of the serious game Dojo, a biofeedback preventive game targeting anxiety among teenagers (65). Results revealed generalization of acquired knowledge and reduction of anxiety levels, but the decrease of symptoms was equal both in the experimental group and the control group (commercial game). During the training, adolescents reported difficulties in maintaining motivation and expressed boredom after approximately four sessions (they finished the game and repeated the same levels over and over during the last two sessions). Other factors could explain the lack of correlation between efficacy of modern serious games and duration of the intervention. We included articles published after 2012 whereas Grynszpan et al. included articles published between 1995 and 2012 (25). Video games, across all platforms (console, tablet or computer) are getting more intuitive and accessible every year and users are getting more comfortable with these new technologies, specifically children and adolescents. Supervision is no longer necessary to help participants familiarize with these technologies expected for specific hardware-based games such as biofeedback, neurofeedback and specifically designed serious exergames.

Modern serious games can also include online options. Six of the 14 serious games retrieved in this literature review can be played online. Online games can allow the psychiatrist to remotely supervise the patient and monitor his progress as well as to adapt goals of the game, making supervision during intervention less necessary. Another benefit of online serious game is their ability to stimulate pro social behavior and competition by connecting player with each other through internet, thus increasing the estimated playing time of the game while increasing motivation.

The game “plan it commander”, a serious game for children with ADHD had the longest intervention in our literature review (10 weeks) and required to be played three times a week for 65 min (48). The game managed to maintain a high level of motivation by enabling players to communicate with messages and compete with one another through rankings and leaderboards. Results of the evaluation of the efficacy of this serious game showed positive results with significant improvement on time management, social skills and working memory (48).

Most of the commercially available video games retrieved in this literature review were exergames (five out of eight). Exergames can target somatic symptoms that can often be part of child and adolescent psychiatric disorders or treat symptoms through physical exercises. Exergames thus increase the range of psychiatric disorders treated by video games by targeting symptoms that are not addressed by sedentary games. They were the only games found in this systematic review to be used as part of the treatment of dyslexia and developmental coordination disorder.

Studies evaluating commercial video games (exergames and sedentary games) did not benefit of the same improvement in quality during the past few years in comparison to serious games. These studies included less participants (18 to 100) and suffered from the same limitations as those evaluating serious games (none of them uses a gold standard therapy as control group nor includes any evaluation after intervention and six studies did not include a waitlist). Overall, these games show less generalization (three out of eight studies did not show any generalization or any improvement on targeted symptoms).

A majority of studies refer to specific advantages that serious games can hold for therapeutic use in child and adolescent psychiatry. All studies revealed that these games were well received by children and teenagers and dropout rates were low. These games can represent potential alternatives to usual care for adolescents, as pointed out by the most rigorous RCTs in our selection, those evaluating SPARX (42) and Mindlight (41). These studies showed non inferior results to treatment-as-usual for mild-to-moderate depression in adolescents, and anxiety in children. The main variation, in comparison to treatment-as-usual, is that they require minimal intervention from a clinical practitioner: Mindlight was accessible at school in groups of five-to-ten students supervised by two master students and SPARX was accessible from home without any supervision. Additionally, there is a need for alternative ways of delivering psychotherapy since psychiatric pathologies and therapies can hold a certain stigma, and that it can be difficult for youth in need of mental health care to access proper treatment for geographic or financial reasons (69). SPARX could become a relevant self-help resource, overcoming all those barriers by being easily accessible at home, through internet. Thanks to these characteristics, serious games opened new doors in preventive child and adolescent psychiatry, as shown by the two studies evaluating most thoroughly these games among the retrieved studies. REThink (40) was used to prevent emotional disorders in children and adolescents and SPARX (70) to prevent depression in final-year secondary students. Both studies showed results that compare favorably with CBT equivalent based prevention programs, but again, these games are cheaper than standard CBT, easily accessible and easy to disseminate in schools, making them cost-effective tools for child and adolescent psychiatry prevention programs.

Despite these advantages, to this day serious game included in this literature review are mainly limited to research-based field.

In line with Grynszpan et al., we consider that these innovative games should be accessible for parents and educators (25), ideally by making their purchase available online for a reasonable fee. By becoming available on internet, serious games could reach a substantial population and benefit from users' feedbacks that could help in return developers to improve them.

## Conclusion

Serious games can now be considered as an innovative adjunct or alternative in the treatment and prevention of child and adolescent depression and anxiety disorders but also as part of the treatment of ADHD and ASD. In recent years, many well-conducted randomized controlled trials have been published demonstrating promising results. Efforts should be made to make these high quality serious games available to parents and educator.

Future research evaluating serious games and commercially available games (specifically exergames) as part of the treatment of child and adolescent psychiatric disorders should include comparison to a control group undergoing face to face gold standard psychotherapy, a waitlist group and long terms follow-up evaluation. This literature review also highlighted that commercially available exergames hold important promise as a powerful new therapeutic and preventive tool in the treatment of developmental coordination disorder and autism spectrum disorder.

## Author Contributions

DZ and AR designed the literature search and wrote the protocol. DZ and AR performed the literature search. DZ and AR analyzed the data. DZ, J-PR, and AR contributed to the interpretation of the data. DZ wrote the first draft of the manuscript and AR and J-PR critically revised the manuscript for important intellectual content. All authors contributed to and have approved the final manuscript.

## Conflict of Interest

The authors declare that the research was conducted in the absence of any commercial or financial relationships that could be construed as a potential conflict of interest.
